# Two-Week Central Macular Thickness Reduction Rate >37% Predicts the Long-Term Efficacy of Anti-vascular Endothelial Growth Factor Treatment for Macular Edema Secondary to Retinal Vein Occlusion

**DOI:** 10.3389/fmed.2022.851238

**Published:** 2022-03-07

**Authors:** Jialin Zhou, Huafeng Ma, Xiyuan Zhou, Qiuyu Wang, Weihou Li, Shuai Luo, Chang Cai, Zefeng Li, Danning Liu

**Affiliations:** Department of Ophthalmology, Second Affiliated Hospital of Chongqing Medical University, Chongqing, China

**Keywords:** retinal vein occlusion (RVO), macular edema (ME), anti-vascular endothelial growth factor (anti-VEGF), early response assessments, central macular thickness (CMT)

## Abstract

**Objective:**

To determine if the early response assessments can predict the long-term efficacy of anti-vascular endothelial growth factor (VEGF) treatment for macular edema secondary to retinal vein occlusion (RVO-ME).

**Methods:**

A retrospective study of patients with diagnosis of RVO-ME and intravitreal anti-VEGF treatment was conducted. Clinical characteristics including age, gender, disease subtype and disease duration were recorded at baseline. The best corrected visual acuity (BCVA and logMAR), intraocular pressure (IOP), and central macular thickness (CMT) were recorded at baseline, 2 weeks, and every month (months 1–6) after injection. Further, we compared the early response assessments between the cured group (6-month CMT ≤ 250 μm) and the uncured group (6-month CMT > 250 μm).

**Results:**

A total of 164 eyes in 164 patients (77 male and 87 female) were included. At each post-injection time point, both BCVA and CMT are significantly decreased from baseline (all *P* < 0.001). Spearman’s test showed that 2-week CMT reduction rate after the first injection was negatively correlated with BCVA at 6 months (*r* = −0.359, *P* < 0.001). Compared with the uncured group (47 cases), the cured group (117 cases) was younger (59.53 ± 11.68 vs. 65.19 ± 13.10 years old, *P* < 0.01), had more BRVO patients (76.1% vs. 44.7%, *P* < 0.01), a shorter disease duration (1.92 ± 2.43 vs. 5.05 ± 4.32 months, *P* < 0.01), lower baseline CMT (527.09 ± 154.95 vs. 768.96 ± 287.75 μm, *P* < 0.01), and lower baseline BCVA (0.86 ± 0.44 vs. 1.31 ± 0.51, *P* < 0.01). At each post-injection time point, the cured group had lower CMT and BCVA values when compared to the uncured group (all *P* < 0.01), and the 2-week CMT reduction rate was identified as the earliest response time to predict the long-term treatment efficacy. Moreover, ROC curve analysis indicated that a 2-week CMT reduction rate >37% yielded the best cut-off point for predicting the long-term cure of anti-VEGF treatment at 6 months (*P* < 0.001). Multivariable logistic regression confirmed that the 2-week CMT reduction rate >37% was independently associated with the 6-month cured rate (OR = 9.639, 95% Cl = 1.030–90.227, *P* = 0.047).

**Conclusion:**

Age, disease duration, baseline CMT, and baseline BCVA are associated with visual outcomes at 6-month of anti-VEGF treatment for RVO-ME. The “2-week CMT reduction rate >37%” after the first injection is an independent factor to predict better long-term outcomes.

## Introduction

Retinal vein occlusion (RVO) is the second most blinded retinal vascular disease in the world (1), which is characterized with the blockage of small veins that carries blood away from the retina. This blockage is often associated with the hardening of arteries (such as atherosclerosis) and the formation of a blood clot (2). Risk factors for RVO include older age, active smoking, hypertension, diabetes mellitus type II, hyperhomocysteinemia, dyslipidemia, carotid artery disease, glaucoma, and obstructive sleep apnea syndrome (3–5). The two most common types of RVO are central retinal vein occlusion (CRVO) and branch retinal vein occlusion (BRVO) according to where the occlusion is located. Though the pathogenesis is different, they both may cause retinal ischemia which gives rise to up-regulation of some cytokines such as vascular endothelial growth factor (VEGF), placental growth factor (PlGF), etc., and further lead to macular edema (ME) (6). Notably, ME, the most common complication of RVO, often results in decreased vision and even blindness, which seriously influences the prognosis of patients (7). Recent RVO guidelines have recommend intravitreal injection of anti-VEGF drugs as the first-line treatment for RVO-ME, due to its ability to inhibit angiogenesis, reduce vascular permeability, relieve inflammation, thus reducing central macular thickness (CMT) and ME (6–8). In clinics, the anti-VEGF drugs are mainly divided into the monoclonal antibody (mAb, such as ranibizumab) and the Fc-fusion protein (Fc, such as aflibercept, conbercept, etc.). However, some patients continue to have ME or relapse after repeated injection in clinic practice, namely refractory ME, which requires long-term, repeated, and combined treatment (9, 10).

Optical coherence tomography (OCT) is one of the commonly used detection techniques in ophthalmology. It can accurately measure CMT which is a key index for evaluating the efficacy of ME treatment and determining if repeated injection is necessary. Previous studies have found that visual function increases the most at 7 days after anti-VEGF treatment (11–13). While recent studies on BRVO state that CMT decreases most 24 h after the first anti-VEGF treatment, and it can be utilized as a prognostic factor for long-term efficacy (14). Similar investigation is limited and current studies have variable standards to determine the treatment efficacy of patients (15, 16).

Therefore, this study aimed to investigate if the early response assessments such as the best corrected visual acuity (BCVA), intraocular pressure (IOP), CMT value, and CMT reduction rate, can predict the long-term efficacy of anti-VEGF treatment for RVO-ME. We are also interested in the comparison of the long-term efficacy between the monoclonal antibody (mAb) and Fc-fusion protein (Fc)-based anti-VEGF drugs. The receiver operating characteristic (ROC) curve plot is utilized to illustrate the best classifier of early CMT reduction rate to predict the long-term efficacy of anti-VEGF treatment. In this way, refractory ME can be found early in the clinic and provide evidence for individualized therapy.

## Materials and Methods

### Study Design and Participants

**FIGURE 1:**
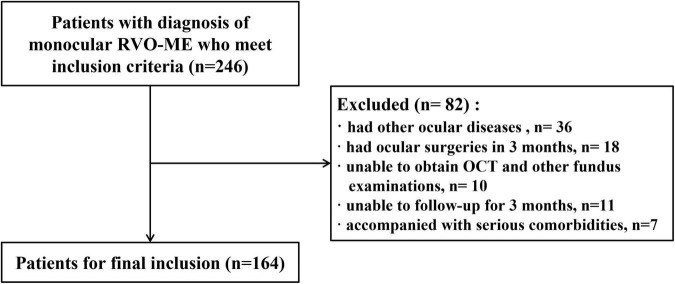
The flow chart of enrolled patients.

This retrospective study was conducted at Ophthalmology Department of the Second Affiliated Hospital of Chongqing Medical University from July 2017 to January 2021 in accordance with the Declaration of Helsinki. The local institutional ethics review boards approved the study’s protocol (number 2018-56) and written informed consent was obtained from all patients. The inclusion criteria were as follows (13): (1) Patients were ≥ 18 years old and had disease duration (the time from the onset of RVO-related symptoms to the first anti-VEGF treatment) ≤ 1year. (2) Patients were diagnosed with macular edema secondary to CRVO or BRVO with the central macular thickness (CMT) > 250 μm per OCT examination. (3) The baseline of the best corrected visual acuity (BCVA, logMAR) fell into the range of 0.3–1.0 (6–8). (4) Patients underwent multiple intravitreal injections of anti-VEGF drugs (mAb or Fc-binds) alone by the same surgeon. The exclusion criteria (13) were patients who had other ocular diseases such as severe cataracts, glaucoma, uveitis, high myopia (over −6.00D), and macular diseases, or had ocular surgeries within 3 months before the first anti-VEGF injection such as retinal photocoagulation, vitrectomy and vitreous injection. Patients who experienced a change in anti-VEGF agent or underwent retinal photocoagulation after baseline were excluded. Patients who were unable to obtain OCT and other fundus examinations due to refractive stroma turbidity, unable to follow-up for 3 months, and accompanied with serious comorbidities such as stroke and myocardial infarction were excluded. The flow chart of enrolled patients was showed in Figure 1.

### Materials and Instruments

Anti-vascular endothelial growth factor drugs used in our hospital include ranibizumab (Swiss Novartis), conbercept (Chengdu Kanghong Biotechnology Co., Ltd.), and aflibercept (Germany Bayer). IOP was measured by Full Auto Tonometer (Canon, Japan) and central macular thickness (CMT) was measured in a 512*128 mode by Cirrus HD-OCT 5000 (Zeiss, Germany).

### Data Collection, Treatment, and Follow-Up

The patients’ characteristics including gender, age, disease subtype, disease duration, BCVA (logMAR), CMT, IOP, and other baseline indexes were retrospectively reviewed and retrieved. LogMAR is a notation of vision loss. Zero logMAR indicates standard vision, positive values indicate poor vision, and negative values indicate good visions. The patients were qualified for repeated anti-VEGF injection only when they met such criteria: persisted macular edema or recurrent worse macular edema (CMT > 250 μm), or intraretinal fluids/subretinal fluids was visible in OCT (12, 17). All patients’ monocular eyes were treated with anti-VEGF drugs intravitreally in accord with the principle of first-time and on-demand treatment (1+PRN). The first follow-up time was 2-weeks after the first injection, followed by 1 to 6 months after injection (once a month). At each follow-up time point, BCVA, IOP, CMT, and the adverse reactions were recorded. Macular edema was defined as CMT > 250 μm, the cure of macular edema was defined as CMT ≤ 250 μm, and the CMT reduction rate was defined below (14). There should be at least a 4-week gap between the adjacent two injections.


$$\begin{array}{c} \mathrm{CMT}\,\mathrm{reduction}\,\mathrm{rate}=\frac{{\text{CMT}}_{\mathrm{before}\,\mathrm{treatment}}-{\text{CMT}}_{\mathrm{after}\,\mathrm{treatment}}}{{\text{CMT}}_{\mathrm{before}\,\mathrm{treatment}}} \end{array}$$

### Comparison of the Efficacy of Different Anti-vascular Endothelial Growth Factor Drugs

The anti-VEGF agents used in the study were classified into two groups based on their different mechanisms of action. They were the monoclonal antibody (mAb) group (injection of ranibizumab, 95 eyes) and Fc-fusion protein (Fc) group (injection of conbercept or aflibercept, 69 eyes). The two groups were compared at baselines such as age, disease subtype, disease duration, BCVA (logMAR), IOP, and CMT, and changes of CMT and BCVA (logMAR) against baselines at each observation time point after treatment. The number of injections was also compared between the two groups.

### The Relationship Between Early Response Assessments and Long-Term Efficacy After Anti-vascular Endothelial Growth Factor Treatment

To find the earliest time possible with the most change of BCVA (logMAR) and CMT, the alternation of BCVA (logMAR) and CMT relative to the baseline among 2-weeks after treatment, 1–6 months after treatment were compared. Spearman correlation analysis was used to evaluate the correlation between the baseline indexes, the early response assessments, and the prognosis of BCVA (logMAR) after 6 months. The strength of the correlation was defined by the absolute value of the correlation coefficient: 0.8–1.0 as very strong correlation, 0.6–0.8 as strong correlation, 0.4–0.6 as moderate correlation, 0.2–0.4 as weak correlation, and 0.0–0.2 as extremely weak or no correlation. The patients were then divided into the cured group (CMT ≤ 250 μm) and the uncured group (CMT > 250 μm) based on their CMT measurement at the 6-month follow-up time. Then the baseline indexes, the early response assessments, and the long-term efficacy via the ROC curve were compared between the two groups. Finally, the early CMT reduction rate threshold that can predict the long-term cure in 6-month were determined and its predictive value in 6-month was further verified by using the multivariable logistic regression analysis.

### Statistical Analysis

The statistical analysis was performed by the SPSS 26.0 software (IBM/SPSS, Chicago, IL, United States). The continuous variables are described as mean ± SD. An independent-sample t-test was used for comparison between the two groups. A paired-sample t-test was used for comparison before and after treatment. The enumeration variables were described as *n* (%) and were compared by a Chi-square test. Spearman’s test and multivariable logistic regression analysis were used to analyze the correlation between the early response assessments of the affected eye and long-term efficacy following anti-VEGF treatment for RVO-ME. The receiver operating characteristic (ROC) curve was used to determine the early CMT deduction rate threshold for predicting long-term efficacy at 6 months. All tests were two-side and *P* < 0.05 was considered statistically significant.

## Results

### Baseline Characteristics

This study included 164 eyes in 164 RVO patients, of whom 77 were male and 87 were female. The age was 61.15 ± 12.33 years old. A total of 110 patients were diagnosed with BRVO, and 54 patients were diagnosed with CRVO. The disease duration were2.82 ± 3.39 months, the baseline BCVA (logMAR) was 0.99 ± 0.51, and the baseline CMT was 596.41 ± 229.10 μm (Table 1). No intraocular adverse events such as increased IOP, vitreous hemorrhage, and endophthalmitis are observed in all patients. During the study period, there were no patients who experienced a change in anti-VEGF agent or underwent retinal photocoagulation.

**TABLE 1:** Demographic characteristics of patients.

Characteristics	Mean ± SD
Age (years)	61.15 ± 12.33
Disease duration (month)	2.82 ± 3.39
Baseline of best corrected visual acuity (logMAR)	0.99 ± 0.51
Baseline of central macular thickness (μm)	596.41 ± 229.10
Preoperative intraocular pressure (mmHg)	15.12 ± 5.76

### Comparison of the Efficacy of Different Anti-vascular Endothelial Growth Factor Drugs

**FIGURE 2:**
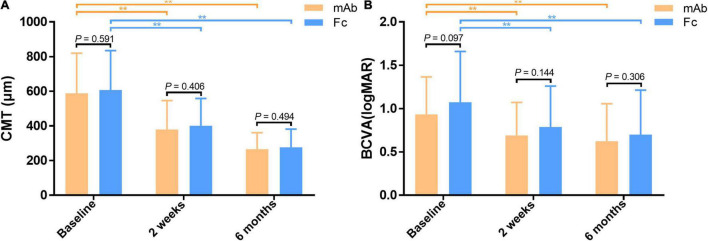
**(A)** Comparison of CMT between the monoclonal antibody-treated group and the Fc-fusion protein-treated group. **(B)** Comparison of BCVA between the monoclonal antibody-treated group and the Fc-fusion protein-treated group. CMT and BCVA (logMAR) are significantly decreased from the baseline in both groups at half-month and 6 months after treatment. **Significant difference compared to the baseline (*P* < 0.01). However, there is no significant difference in mean CMT and BCVA (logMAR) between the two groups at half-month and 6 months post-treatment (*P* > 0.05). BCVA, best corrected visual acuity (logMAR). CMT, central macular thickness (μm).

There were no significant differences in terms of gender, age, disease subtype, disease duration, BCVA (logMAR), IOP, CMT, and the number of injections between the mAb group and the Fc group (*P* > 0.05) (Table 2). The CMT and BCVA (logMAR) within the two groups are significantly reduced than the baseline at each observation time after anti-VEGF treatment, indicating a statistically significant difference (*P* < 0.01); while the comparison between the two groups showed no statistical difference (*P* > 0.05) (Figure 2). According to the criteria of repeated injection, 65.26% (62/95 eyes) of patients in the mAb group had repeated injections, and 23.16% (22/95 eyes) had no recurrence followed up for 6 months after the first injection. The average injection was 2.21 ± 1.12 times. In comparison, 57.97% (40/69 eyes) of patients in the Fc group had repeated injections, and 24.64% (17/69 eyes) had no recurrence followed up for 6 months after the first injection. The average injection had 2.23 ± 1.30 times. The injection times between the two groups were not statistically significant (*t* = −0.113, *P* = 0.910). All patients had significantly decreased CMT and improved vision following anti-VEGF treatment in both mAb and Fc group. There was no significant difference in terms of the efficacy and the number of injections between the two different drugs.

**TABLE 2:** Comparison the baseline between the monoclonal antibody group and the Fc-fusion protein group.

Characteristics	Monoclonal antibody group (*n* = 95)	Fc-fusion protein group (*n* = 69)	χ^2^/t	*P* value
Age (years old)	61.48 ± 11.70	60.70 ± 13.23	0.403	0.687
**Gender, *n* (%)**				
Male	43 (45.3%)	34 (49.3%)	0.258	0.611
Female	52 (54.7%)	35 (50.7%)		
**Disease subtype, *n* (%)**				
BRVO	63 (66.3%)	47 (68.1%)	0.059	0.809
CRVO	32 (33.7%)	22 (31.9%)		
Disease duration (month)	2.79 ± 3.41	2.85 ± 3.38	–0.105	0.917
Baseline BCVA (logMAR)	0.93 ± 0.43	1.07 ± 0.59	–1.673	0.097
Baseline CMT	588.19 ± 231.71	607.72 ± 226.66	–0.538	0.591
Baseline IOP (mmHg)	15.69 ± 7.24	14.34 ± 2.44	1.482	0.140
Number of injections	2.21 ± 1.12	2.23 ± 1.30	–0.113	0.910

*BRVO, branch retinal vein occlusion; CRVO, central retinal vein occlusion; BCVA, best corrected visual acuity; CMT, central macular thickness; IOP, intraocular pressure. There are no significant differences in terms of gender, age, disease subtype, disease duration, BCVA (logMAR), IOP, CMT, and the number of injections between the mAb group and the Fc group (P > 0.05).*

### The Relationship Between Early Response Assessments and Long-Term Efficacy After Anti-vascular Endothelial Growth Factor Treatment

#### Determination of the Early Response Time

**FIGURE 3:**
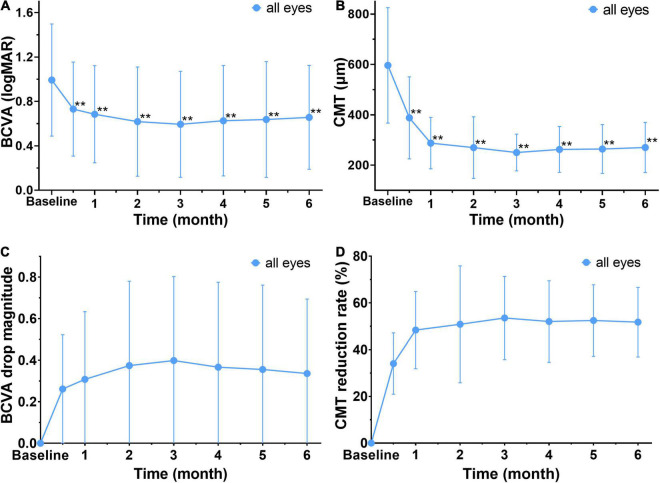
Changes in mean BCVA and CMT before and after treatment in all affected eyes. **(A,B)** The mean BCVA (logMAR) and CMT are decreased significantly after treatment when compared to baseline. **Significant different compared to baseline (*P* < 0.01). **(C)** The drop magnitude of mean BCVA (logMAR) is compared to baseline at each observation time point after treatment, and it shows a notable decrease at 2 weeks after the first treatment with a change of 0.34 ± 0.36. **(D)** The drop rate of CMT at each observation time after treatment. It shows a notable reduction of CMT at 2 weeks after the first treatment, with a drop rate 34 ± 13 %. BCVA, best corrected visual acuity (logMAR). CMT, central macular thickness (μm).

The patients were analyzed to investigate the relationship between early response assessments and the long-term efficacy of anti-VEGF treatment. The results showed that the BCVA (logMAR) of the patients was significantly lower than that in the baseline (0.99 ± 0.51) after 2 weeks, 1, 2, 3, 4, 5, and 6 months of treatment (*P* < 0.01) (Figures 3A,C). The CMT of the observed eyes was significantly lower than the baseline after 2 weeks, 1, 2, 3, 4, 5, and 6 months of treatment, and the difference was statistically significant (*P* < 0.01) (Figures 3B,D). Thus, the macular edema was resolved significantly, and vision was improved remarkably after anti-VEGF treatment. Furthermore, the mean CMT and BCVA (logMAR) had decreased the most in 2 weeks post the first injection (Figures 3C,D). Therefore, 2 weeks was the earliest response time for follow-up investigation.

#### Analysis of Indexes That May Affect Long-Term Efficacy

The Spearman correlation analysis was used to analyze the correlation between the baseline, the early follow-up indexes, and the prognosis of BCVA (logMAR) after 6 months. The 6-month BCVA (logMAR) post the first treatment was considered as the outcome to measure prognosis of BCVA.

**FIGURE 4:**
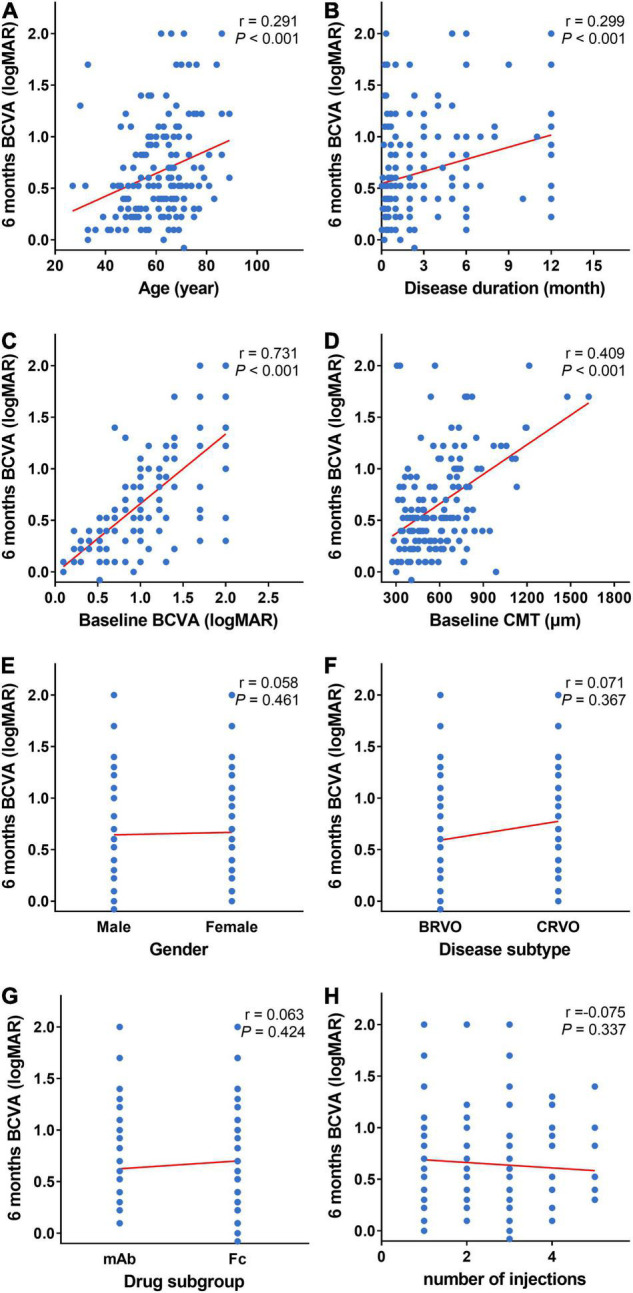
Scatter plots of baseline and 6-month BCVA. **(A–H)** Scatter plots of 6-month BCVA (logMAR) and baseline indexes (age, disease duration, BCVA (logMAR), CMT, gender, disease subtype, and anti-VEGF drug subgroup). Six-month BCVA (logMAR) is strongly correlated with baseline BCVA (logMAR) **(C)**, moderately correlated with baseline CMT **(D),** and weekly correlated with age **(A)** and duration of disease **(B)**. Six-month BCVA (logMAR) is not correlated with gender **(E)**, disease subtype **(F)**, anti-VEGF drug subgroup **(G)**, and the number of injections **(H)**. BCVA: Best corrected visual acuity (logMAR). CMT, central macular thickness (μm); VEGF, vascular endothelial growth factor.

It showed the 6-month BCVA (logMAR) was strongly correlated with the baseline BCVA (logMAR), moderately correlated with the baseline CMT and weakly correlated with the age and disease duration. Moreover, the 6-month BCVA (logMAR) is not correlated with gender, disease subtype, anti-VEGF drug subgroup and the number of injections (Figures 4A–H).

**FIGURE 5:**
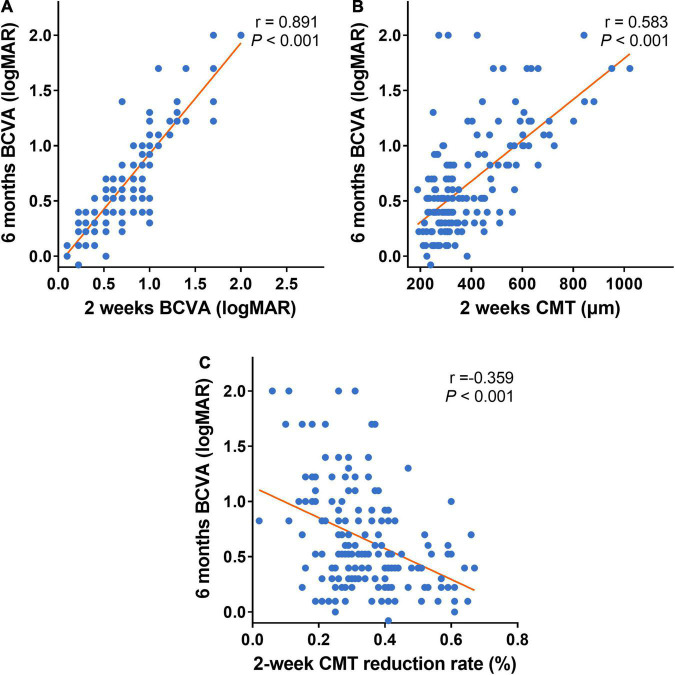
Scatter plots of the early follow-up indexes and 6-month BCVA. **(A–C)** Scatter plots of 6-month BCVA (logMAR) and early follow-up indexes (2-week BCVA (logMAR), 2-week CMT, and 2-week CMT reduction rate). Six-month BCVA (logMAR) is strongly correlated with 2-week BCVA (logMAR) after the first treatment **(A)**. Six-month BCVA (logMAR) is moderately correlated with 2-week CMT after the first treatment **(B)**. Six-month BCVA (logMAR) is weekly correlated with 2-week CMT reduction rate after the first treatment **(C)**. BCVA: Best corrected visual acuity (logMAR). CMT, central macular thickness (μm).

It also showed the 6-month BCVA (logMAR) was strongly correlated with the 2-week BCVA (logMAR) post the first injection, moderately correlated with the 2-week CMT post the first treatment, and weakly correlated with the 2-week CMT reduction rate (Figures 5A–C).

#### Relationship Between Early Response Assessments and Long-Term Efficacy

**FIGURE 6:**
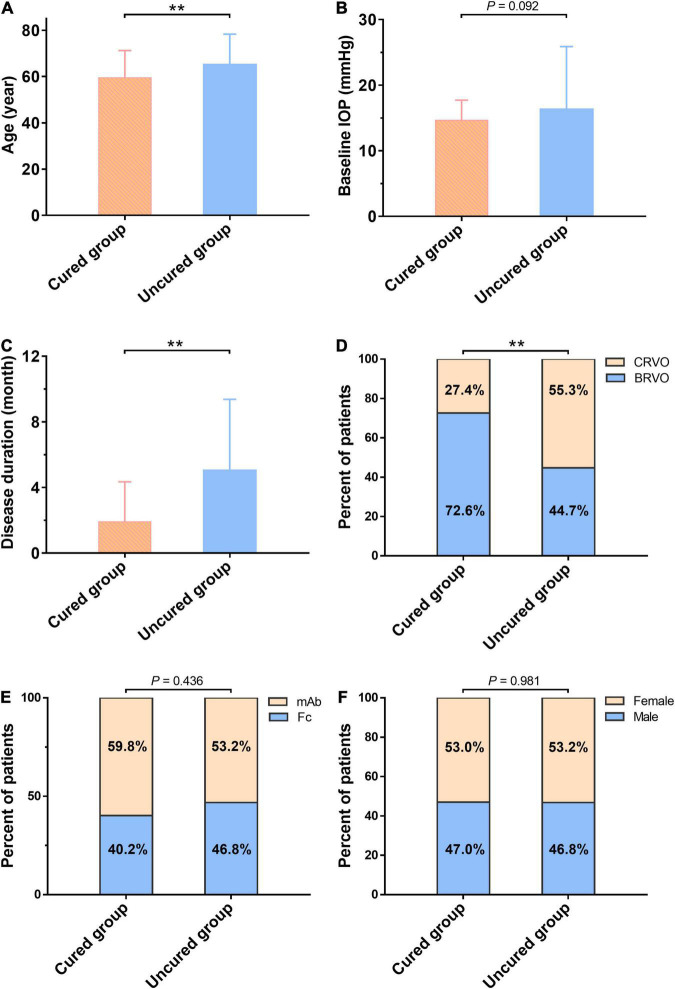
Comparison of baseline characteristics between the cured group and the uncured group at the 6-month follow-up. There is no significant difference in Gender **(F)**, anti-VEGF drug subgroup **(E)**, and IOP **(B)** between the two groups. Comparison of the baseline age reveals the age in the cured group is significantly younger than that of the uncured group (*t* = –2.709, *P* = 0.007) **(A)**. The cured group has 76.1% of BRVO, while the uncured group has 44.7% of BRVO, and the difference is statistically significant (χ^2^ = 14.957, *P* < 0.001) **(D)**. The duration of disease in the cured group is notably shorter than that of the uncured group (*t* = 4.678, *P* < 0.001) **(C)**. **The difference is statistically significant (*P* < 0.01). mAb, the monoclonal antibody-based anti-VEGF drugs; Fc, Fc-fusion protein-based anti-VEGF drugs; BCVA, best corrected visual acuity (logMAR); BRVO, branch retinal vein occlusion; CMT, central macular thickness (μm); IOP, intraocular pressure; VEGF, vascular endothelial growth factor.

The patients were divided into the cured group (CMT ≤ 250 μm, 117 eyes) and the uncured group (CMT > 250 μm, 47 eyes) based on their CMT measurement in OCT at the 6-month follow-up time. There was no significant difference in gender, anti-VEGF drug subgroup, IOP, and the number of injections between the cured and uncured groups. Comparison of the baseline age reveals the age in the cured group was significantly younger than that of the uncured group (*t* = −2.709, *P* = 0.007). The cured group had 76.1% of BRVO, while the uncured group had 44.7 % of BRVO, and the difference was statistically significant (χ^2^ = 14.957, *P* < 0.001). The duration of disease in the cured group was notably shorter than that of the uncured group (*t* = 4.678, *P* < 0.001). Both baseline CMT and baseline BCVA (logMAR) in the uncured group were significantly higher than that of the cured group (*t* = 5.454, 5.588, respectively, both *P* < 0.001) (Figure 6). In summary, the cured group was younger, had a shorter disease duration, a greater proportion of BRVO, lower values of baseline CMT and baseline BCVA (logMAR) when compared to the uncured group.

**FIGURE 7:**
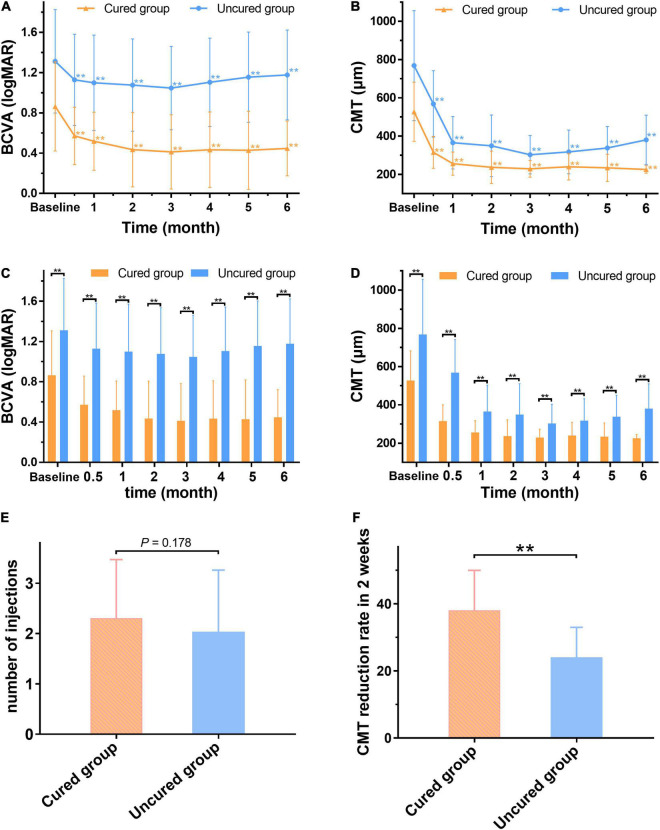
The changes of BCVA and CMT between the cured and uncured eyes before and after treatment. After treatment, the mean BCVA (logMAR) and CMT of the two groups are significantly lower than the baseline (*p* < 0.01). **(A,B)** Both mean BCVA (logMAR) and CMT of the cured group and uncured group are significantly lower than those of baseline (*P* < 0.01). **(C,D)** The cured group has significantly lower BCVA (logMAR) and CMT values compared with the uncured group at any time point after treatment (*P* < 0.01). **(E)** There is no significant difference in the number of injections between the cured group and the uncured group (*P* > 0.05). **(F)** The CMT reduction rate in the cured group is significantly higher than that of the uncured group 2 weeks after the first injection (*P* < 0.01). **The difference is statistically significant (*P* < 0.01). BCVA: Best corrected visual acuity (logMAR). CMT: Central macular thickness (μm).

**FIGURE 8:**
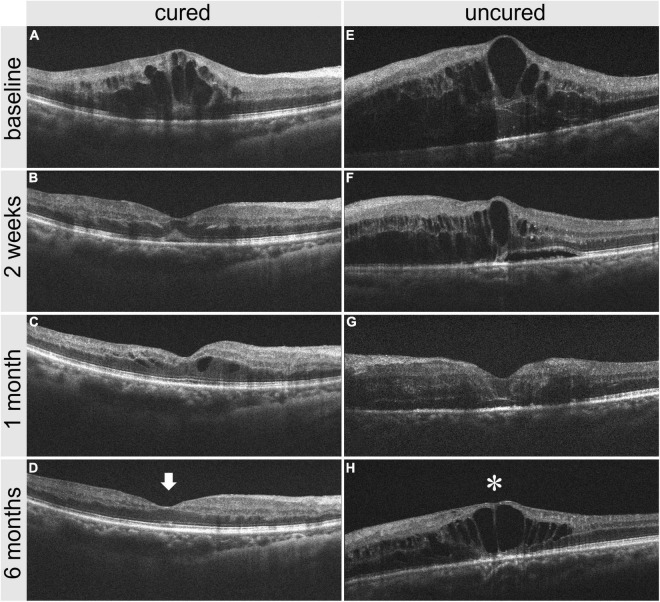
Comparison of OCT images between one cured patient and one uncured patient before and after treatment. **(A–D)** panel shows the OCT images of a cured patient before treatment, 2 weeks, 1 month, and 6 months after treatment. **(E–H)** panel shows the OCT images of an uncured patient before treatment, 2 weeks, 1 month, and 6 months after treatment. It is noted that the CMT reduction in the cured patient is greater than that in the uncured patient 2 weeks after treatment. The CMT in the cured patient is 216 μm (arrow) at 6 months after treatment, versus 541 μm (star) in the uncured patient. CMT, central macular thickness; OCT, optical coherence tomography.

After the first injection of anti-VEGF drugs, the 2-week CMT reduction rate in the cured group was greater than that in the uncured group (*P* < 0.001) (Figure 7F). The CMT and BCVA (logMAR) values in the cured groups were significantly lower than those in the uncured group at each observation time point from 2 weeks to 6 months (*P* < 0.01) (Figures 7A–D). In addition, there was no significant difference in the number of injections between the two groups (*P* > 0.05) (Figure 7E). Taken together, it suggested that the cured group had a higher 2-week CMT reduction rate and vision improvement than the uncured group after the same number of anti-VEGF injections, and the effect was maintained to 6 months after treatment (Figure 8).

#### Receiver Operating Characteristic Curve Plot to Illustrate the Predicting Ability of Early Response Assessments

**FIGURE 9:**
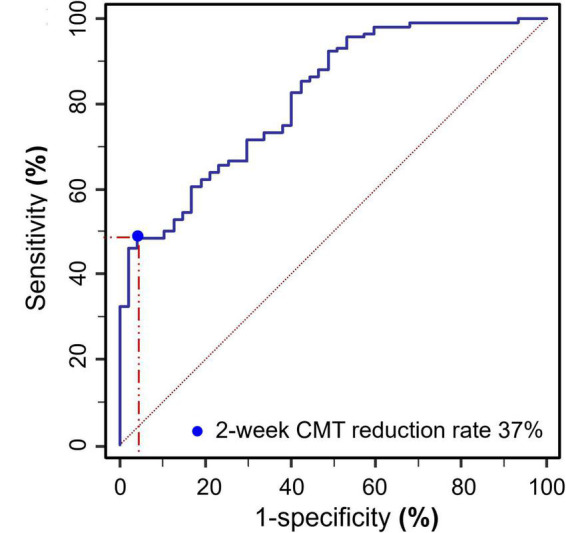
ROC curve plot of the 2-week CMT reduction rate after the first treatment and the cure of RVO patients after 6 months follow up (AUC = 0.816, sensitivity = 95.74%, specificity = 48.72%, Youden index = 0.4446, *P* < 0.001).

Two-week CMT reduction rate after the first treatment was used as the binary classifier to define RVO patients as cured or uncured in 6 months. Then the 2-week CMT reduction rate after the first treatment and the cure of RVO patients after 6 months follow up were utilized to plot the receiver operating characteristic (ROC) curve (Figure 9). The result showed that a 2-week CMT reduction rate >37% after the first treatment was the best classifier to determine the efficacy of anti-VEGF treatment after 6 months (AUC = 0.816, sensitivity = 95.74%, specificity = 48.72%, Youden index = 0.4446, *P* < 0.001).

#### Logistic Regression Analysis

To further verify the predicting ability of 2-week CMT reduction rate for a long-term cure, a multivariable logistic regression analysis was used to predict the relationship between predictors (the independent variables such as age, disease subtype, disease duration, 2-week CMT, 2-week CMT reduction rate, and 2-week BCVA (logMAR)) and a predicted variable (the dependent variable: cure of RVO patients at 6 months). The results revealed that the 2-week CMT reduction rate >37% after the first injection was the best predictor for the cure at 6 months (OR = 9.639, 95% Cl = 1.030–90.227, *P* = 0.047). In addition, a longer disease duration (OR = 0.675, 95% Cl = 0.546–0.835, *P* < 0.001), a higher 2-week CMT value (OR = 0.982, 95% Cl = 0.974–0.990, *P* < 0.001), and a higher 2-week BCVA (logMAR) (OR = 0.087, 95% Cl = 0.012–0.609, *P* = 0.014), were negative predictors for the cure of RVO at 6 months (Table 3).

**TABLE 3:** Logistic regression analysis of indexes that may affect the cure of RVO at 6 months.

Risk index	β	OR	95% confidence interval (CI)		*P* value
			Lower limit	Upper limit	
Age	–0.060	0.942	0.880	1.007	0.080
Disease subtype	–0.488	0.614	0.145	2.609	0.509
Disease duration	–0.393	0.675	0.546	0.835	< 0.001
2-week CMT	–0.018	0.982	0.974	0.990	< 0.001
2-week CMT reduction rate >37%	2.266	9.639	1.030	90.227	0.047
2-week BCVA (logMAR)	–2.443	0.087	0.012	0.609	0.014

*During the effect risk index (P < 0.05), 2-week CMT reduction rate >37% is the best predictor for the cure at 6 months (OR = 9.639, 95% Cl = 1.030–90.227, P = 0.047). In addition, disease duration, 2-week CMT and 2-week BCVA (logMAR) are negative predictors for the cure of RVO at 6 months.*

## Discussion

Previously most studies focus on utilizing the loss of baseline indexes such as CMT, disorganization of the retinal inner layer (DRIL), subretinal fluid (SRF), hyperreflective foci (HF), external limiting membrane (ELM), ellipsoid zone (EZ), and interdigitation zone (IZ) to predict the long-term efficacy in RVO-ME (10, 17–21). However, due to blockade of retinal hemorrhage before treatment, large CMT, and poor ocular fixation due to poor visual function, the quality of the retinal interlayer structure obtained from OCT images is far from satisfactory. Therefore, using these baseline retinal interlayer structure parameters to predict the long-term efficacy is limited (22). Recent studies have reported that the RVO patients who respond well to anti-VEGF therapy in the early stage are expected to have a better visual function recovery and less burden of long-term treatment. Hoeh et al. (23) have treated 27 CRVO-ME patients with anti-VEGF treatment, and have found 78% of patients who have no recurrence of ME at 6 months have completely resolved ME in 6 weeks after the first injection. Wang et al. (16) have found that early responders (CMT < 320 μm at 3 months after the first injection of anti-VEGF drugs) can achieve better visual improvement than those late or incomplete responders (CMT ≥ 320 μm at 3 months after injection). Bhisitkul et al. (15) did a post-mortem analysis of the BRAVO and CRUISE trials and have found about three-quarters of CRVO patients had CMT dropped to 250 μm or less at 3 months after the first anti-VEGF treatment and they had a better vision at 6–12 months follow-up. Jiang et al. (14) have advanced the detection time to 24 h after injection and have found that BRVO patients whose CMT reduction rate is greater than 18% at 24 h after the first injection will have a better visual prognosis at the 1-year follow-up. However, this study does not include CRVO patients.

Our study is the first one, to the best of our knowledge, to explore the correlation between the early changes of CMT and the long-term prognosis of all types of RVO-ME following anti-VEGF treatment. The results reveal that after 6 months of anti-VEGF therapy (1+PRN principle) in RVO-ME patients, BCVA is significantly improved and CMT is significantly reduced. The 2-week CMT reduction rate after the first injection can be used as an early response assessment to predict the long-term efficacy of anti-VEGF treatment. At 2-week, the CMT and BCVA have the largest magnitudes of reduction and the degree of improvement gradually gets stabilized. No significant difference is found between the mAb and Fc-based anti-VEGF treatment in terms of the efficacy of RVO-ME. Further correlation analysis reveals the CMT reduction rate at 2 weeks after the first injection is significantly correlated with better BCVA at 6 months. Moreover, the CMT reduction rate at 2 weeks in the cured group is significantly higher than that in the uncured group. These findings provide evidence that early response assessments can predict the long-term efficacy of anti-VEGF treatment. In addition, the ROC curve analysis suggests when the 2-week CMT drop rate is greater than 37%, the patient is more likely to achieve a long-term cure, with an AUC of 0.816 (the closer the AUC is to 1, the higher the accuracy rate). This suggests that a 2-week CMT reduction rate >37% after the first treatment is the best classifier to determine the cure of anti-VEGF treatment after 6 months. To further verify the predicting ability of 2-week CMT reduction rate for a long-term cure, the binary logistic regression analysis is used to predict the relationship between the independent variables such as age, disease subtype, disease duration, 2-week CMT, 2-week CMT reduction rate, and 2-week BCVA, and a predicted variable which is the cure of RVO patients at 6 months. We find that the 2-week CMT reduction rate >37% after the first injection is the best predictor for a cure at 6 months. Therefore, the early CMT reduction rate after anti-VEGF treatment can serve as one of the simple and reliable methods to predict the long-term efficacy of RVO-ME. In conclusion, the 2-week CMT reduction rate >37% is a predictor for long-term cure following anti-VEGF treatment. The “2-week CMT reduction rate >37%” will serve as the quantitative evaluation standard to predict the outcome of anti-VEGF therapy and help to guide the individualized treatment for RVO-ME.

Why early CMT reduction rate could predict the low-term efficacy of anti-VEGF treatment? The aquaporin water channels in the retinal Müller glial cells and retinal pigment epithelium (RPE) cells contribute greatly to eliminating ME (24). A high level of VEGF caused by ischemia increases vascular permeability, promotes the release of inflammatory factors and white blood cell adhesion, and slows the blood flow. This in return causes a positive feedback loop that continues to worsen ischemia and bring forth dysfunction and apoptosis of Müller cell and RPE cells (25). The anti-VEGF drugs can block this loop, thereby boosting the aquaporin water channels of residual Müller cells and RPE cells to eliminate ME (25). We are speculating that the difference in early CMT reduction rate may reflect the different degrees of damage to the aquaporin water channels in different patients. In this study, patients whose 2-week CMT reduction rate >37% may have less damage and greater recovery potential of the aquaporin water channels. Therefore, these patients will have a higher rate of long-term cure.

In addition, our study has found other baseline indexes that are correlated with long-term efficacy following anti-VEGF treatment. Spearman’s analysis shows that baseline indexes such as age, disease duration, baseline BCVA, and baseline CMT are significantly positively correlated with the endpoint BCVA. The included patients are divided into a cured group (6 months CMT ≤ 250 μm) and an uncured group (6 months CMT > 250 μm) based on their 6 months CMT values. The comparison between the two groups shows that the cured group is younger, has a shorter disease duration, has a greater proportion of BRVO, and has lower values of baseline CMT and baseline BCVA when compared with the uncured group. This is consistent with previous studies investigating the baseline indexes to predict the long-term efficacy in RVO-ME (26–30). Scott et al. (31) have treated RVO-ME patients with anti-VEGF treatments for six consecutive (once a month) months, and the univariate regression analysis shows that the baseline BCVA and the baseline CMT are significantly correlated with 6-month BCVA. Those patients who have a poor end-point visual acuity usually have a poor baseline BCVA and a high CMT value. In the multivariate model of this study, age and baseline BCVA can independently predict the treatment efficacy. In other studies, age and disease duration are baseline factors that can affect visual function following anti-VEGF treatment (16, 31). The eyes of ME secondary to BRVO are showing a better visual prognosis compared with CRVO (32, 33). In this study, younger patients with a shorter disease duration are associated with better visual outcomes. This may be due to the elderly patients being more likely to have ischemic RVO than younger patients, and younger patients have intact and healthy photoreceptors which hold greater potential for recovery after acute injury (34). Further, some studies have demonstrated that in patients with a long duration of disease, the damage to the photoreceptor and other retinal structures is irreversible. Even when ME is completely dissolved, the long-term visual outcome may be poor (17, 33, 35). This is consistent with our research results. More importantly, the eyes with ME secondary to BRVO have better long-term vision compared with eyes in CRVO at each follow-up time in our study. This confirms the finding of a previous study that the more extensive damage of retinal venous circulation the more severe retinal damage (36).

Our research results also reveal that there is no significant difference between the mAb and Fc-based anti-VEGF treatment in terms of the efficacy of RVO-ME. The anti-VEGF drugs have been widely used in ocular diseases such as exudative age-related macular degeneration, diabetic macular edema, choroidal neovascularization, and RVO-ME, and have shown good safety and efficacy. Previous retrospective and prospective studies have shown that the anti-VEGF treatment for RVO-ME in real-word has achieved significant retinal structure improvement and visual function recovery (5, 17, 29, 33, 37, 38). The patients included in this study are divided into the mAb group (ranibizumab injection) and the Fc group (injection of conbercept or aflibercept) according to their mechanism of action. At the end of the 6-month follow-up, the mean CMT is significantly decreased (*P* < 0.001), and the mean BCVA is significantly improved (*P* < 0.001) in both groups. There is no statistical difference in the mean CMT, BCVA, and the number of injections at any follow-up time between the two groups. It is known that mAb mainly binds to VEGF-A and Fc binds to multiple sites such as VEGF-A, VEGF-B, PlGF, etc (25). The binding affinity of Fc-fusion proteins to VEGF-A is higher than that of mAb, as a result, it is generally considered Fc is more effective to treat RVO-ME than mAb. However, mAbs have a smaller molecular weight compared with Fc-fusion proteins, hence it will enhance their permeability and offset the defect of lower affinity to some extent. Previous clinical trials and the meta-analysis have shown that mAb and Fc-based anti-VEGF treatment have equivalent effects to RVO-ME (37–39), which is consistent with our results.

There are limitations of this study. Our study has a relatively short-term follow-up time and a small sample size. Thereby largescale, high-quality multi-center randomized controlled trials are warranted for stronger evidence based on the conclusion we made here.

## Conclusion

Intravitreal injection of anti-VEGF drugs is a safe and effective avenue for RVO-ME, and mAb and Fc-based anti-VEGF drugs have equivalent effects. Younger patients, a shorter disease duration, lower baseline CMT, and better baseline BCVA are associated with better visual outcomes at 6-month. BRVO-ME has a better visual outcome than CRVO-ME. The early assessment of CMT can predict the long-term efficacy of anti-VEGF drugs. The 2-week CMT reduction rate >37% predicts the long-term cure of anti-VEGF treatment for RVO-ME.

## Data Availability Statement

The original contributions presented in the study are included in the article/supplementary material, further inquiries can be directed to the corresponding author.

## Ethics Statement

The studies involving human participants were reviewed and approved by the Ethics Committee of the Second Affiliated Hospital of Chongqing Medical University. The patients/participants provided their written informed consent to participate in this study.

## Author Contributions

DL, XZ, and HM contributed to the conception and design of the study. JZ, CC, WL, SL, and QW organized the database. JZ and ZL performed the statistical analysis. JZ wrote the first draft of the manuscript. JZ, DL, and ZL wrote sections of the manuscript. All authors contributed to manuscript revision, read, and approved the submitted version.

## Conflict of Interest

The authors declare that the research was conducted in the absence of any commercial or financial relationships that could be construed as a potential conflict of interest.

## Publisher’s Note

All claims expressed in this article are solely those of the authors and do not necessarily represent those of their affiliated organizations, or those of the publisher, the editors and the reviewers. Any product that may be evaluated in this article, or claim that may be made by its manufacturer, is not guaranteed or endorsed by the publisher.

## Abbreviations

BCVA: best corrected visual acuity
BRVO: branch retinal vein occlusion
CMT: central macular thickness
CRVO: central retinal vein occlusion
DRIL: disorganization of retinal inner layer
ELM: external limiting membrane
EZ: ellipsoid zone
Fc: Fc-fusion protein
HF: hyperreflective foci
IOP: intraocular pressure
IZ: interdigitation zone
mAb: monoclonal antibody
ME: macular edema
OCT: optical coherence tomography
PIGF: placental growth factor
ROC: receiver operating characteristic
RVO: retinal vein occlusion
SRF: subretinal fluid
VEGF: vascular endothelial growth factor.

## References

[1] Song P Xu Y Zha M Zhang Y Rudan I. Global epidemiology of retinal vein occlusion: a systematic review and meta-analysis of prevalence, incidence, and risk factors. *J Glob Health.* (2019) 9:010427. 10.7189/jogh.09.010427 31131101 PMC6513508

[2] Stem M Talwar N Comer G Stein J. A longitudinal analysis of risk factors associated with central retinal vein occlusion. *Ophthalmology.* (2013) 120:362–70. 10.1016/j.ophtha.2012.07.080 23177364 PMC3563864

[3] Pacella F Bongiovanni G Malvasi M Trovato Battagliola E Pistone A Scalinci SZ Impact of cardiovascular risk factors on incidence and severity of retinal vein occlusion. *Clin Ter.* (2020) 171:e534–8. 10.7417/ct.2020.2269 33151253

[4] Trovato Battagliola E Pacella F Malvasi M Scalinci SZ Turchetti P Pacella E Risk factors in central retinal vein occlusion: a multi-center case-control study conducted on the Italian population : demographic, environmental, systemic, and ocular factors that increase the risk for major thrombotic events in the retinal venous system. *Eur J Ophthalmol.* (2021). 10.1177/11206721211064469 [Epub ahead of print]. 34854784

[5] Pacella F Pacella E Trovato Battagliola E Malvasi M Scalinci SZ Turchetti P Efficacy and safety of intravitreal fluocinolone acetonide microimplant (ILUVIEN(§)) in patients with chronic diabetic macular edema: 1 year follow-up. *Eur J Ophthalmol.* (2021). 10.1177/11206721211020203 [Epub ahead of print]. 34030511

[6] Pulido J Flaxel C Adelman R Hyman L Folk J Olsen T. Retinal vein occlusions preferred practice pattern(§) guidelines. *Ophthalmology.* (2016) 123:182–208. 10.1016/j.ophtha.2015.10.045 26581559

[7] Schmidt-Erfurth U Garcia-Arumi J Gerendas BS Midena E Sivaprasad S Tadayoni R Guidelines for the management of retinal vein occlusion by the european society of retina specialists (EURETINA). *Ophthalmologica.* (2019) 242:123–62. 10.1159/000502041 31412332

[8] Berger AR Cruess AF Altomare F Chaudhary V Colleaux K Greve M Optimal treatment of retinal vein occlusion: Canadian expert consensus. *Ophthalmologica.* (2015) 234:6–25. 10.1159/000381357 26088287

[9] Tsuboi K Ishida Y Kamei M. Gap in capillary perfusion on optical coherence tomography angiography associated with persistent macular edema in branch retinal vein occlusion. *Invest Ophthalmol Vis Sci.* (2017) 58:2038–43. 10.1167/iovs.17-21447 28384724

[10] Moon BG Cho AR Kim YN Kim JG. Predictors of refractory macular edema after branch retinal vein occlusion following intravitreal bevacizumab. *Retina.* (2018) 38:1166–74. 10.1097/iae.0000000000001674 28489696

[11] Brown DM Campochiaro PA Singh RP Li Z Gray S Saroj N Ranibizumab for macular edema following central retinal vein occlusion: six-month primary end point results of a phase III study. *Ophthalmology.* (2010) 117: 1124–33.e1. 10.1016/j.ophtha.2010.02.022 20381871

[12] Brown D Campochiaro P Bhisitkul R Ho A Gray S Saroj N Sustained benefits from ranibizumab for macular edema following branch retinal vein occlusion: 12-month outcomes of a phase III study. *Ophthalmology.* (2011) 118:1594–602. 10.1016/j.ophtha.2011.02.022 21684606

[13] Larsen M Waldstein SM Boscia F Gerding H Mones J Tadayoni R Individualized ranibizumab regimen driven by stabilization criteria for central retinal vein occlusion. *Ophthalmology.* (2016) 123:1101–11. 10.1016/j.ophtha.2016.01.011 26896124

[14] Jiang B Liu C Zhang ZY Shi J Xu JQ Sun MH Early response after initial anti-VEGF injection to predict the therapeutic effect on macular edema secondary to branch retinal vein occlusion. *Chin J Optom Ophthalmol Vis Sci.* (2019) 5:362–9. 10.3760/cma.j.issn.1674-845X.2019.05.008 30704229

[15] Bhisitkul RB Campochiaro PA Shapiro H Rubio RG. Predictive value in retinal vein occlusions of early versus late or incomplete ranibizumab response defined by optical coherence tomography. *Ophthalmology.* (2013) 120:1057–63. 10.1016/j.ophtha.2012.11.011 23415775

[16] Wang MZ Feng K Lu Y Qian F Lu XR Zang SW Predictors of short-term outcomes related to central subfield foveal thickness after intravitreal bevacizumab for macular edema due to central retinal vein occlusion. *Int J Ophthalmol.* (2016) 9:86–92. 10.18240/ijo.2016.01.15 26949616 PMC4768500

[17] Tang F Qin X Lu J Song P Li M Ma X. Optical coherence tomography predictors of short-term visual acuity in eyes with macular edema secondary to retinal vein occlusion treated with intravitreal conbercept. *Retina.* (2020) 40:773–85. 10.1097/iae.0000000000002444 30640282

[18] Muraoka Y Tsujikawa A Takahashi A Iida Y Murakami T Ooto S Foveal damage due to subfoveal hemorrhage associated with branch retinal vein occlusion. *PLoS One.* (2015) 10:e0144894. 10.1371/journal.pone.0144894 26661582 PMC4677927

[19] Murakami T Okamoto F Iida M Sugiura Y Okamoto Y Hiraoka T Relationship between metamorphopsia and foveal microstructure in patients with branch retinal vein occlusion and cystoid macular edema. *Graefes Arch Clin Exp Ophthalmol.* (2016) 254:2191–6. 10.1007/s00417-016-3382-2 27169934

[20] Nakano E Ota T Jingami Y Nakata I Hayashi H Yamashiro K. Disorganization of the retinal inner layers after anti-VEGF treatment for macular edema due to branch retinal vein occlusion. *Ophthalmologica.* (2018) 240:229–34. 10.1159/000490809 30089307

[21] Yiu G Welch RJ Wang Y Wang Z Wang PW Haskova Z. Spectral-domain OCT predictors of visual outcomes after ranibizumab treatment for macular edema resulting from retinal vein occlusion. *Ophthalmol Retina.* (2020) 4:67–76. 10.1016/j.oret.2019.08.009 31669329 PMC6944743

[22] Rachima S Hirabayashi K Imai A Iesato Y Murata T. Prediction of post-treatment retinal sensitivity by baseline retinal perfusion density measurements in eyes with branch retinal vein occlusion. *Sci Rep.* (2020) 10:9614. 10.1038/s41598-020-66708-0 32541783 PMC7295767

[23] Hoeh A Ach T Schaal K Scheuerle A Dithmar S. Long-term follow-up of OCT-guided bevacizumab treatment of macular edema due to retinal vein occlusion. *Graefes Arch Clin Exp Ophthalmol.* (2009) 247:1635–41. 10.1007/s00417-009-1151-1 19633982

[24] Pannicke T Ivo Chao T Reisenhofer M Francke M Reichenbach A. Comparative electrophysiology of retinal müller glial cells-a survey on vertebrate species. *Glia.* (2017) 65:533–68. 10.1002/glia.23082 27767232

[25] Noma H Yasuda K Shimura M. Cytokines and pathogenesis of central retinal vein occlusion. *J Clin Med.* (2020) 9:3457. 10.3390/jcm9113457 33121094 PMC7692731

[26] Farinha C Marques JP Almeida E Baltar A Santos AR Melo P Treatment of retinal vein occlusion with ranibizumab in clinical practice: longer-term results and predictive factors of functional outcome. *Ophthalmic Res.* (2015) 55:10–8. 10.1159/000440848 26540281

[27] Yoo JH Ahn J Oh J Cha J Kim SW. Risk factors of recurrence of macular oedema associated with branch retinal vein occlusion after intravitreal bevacizumab injection. *Br J Ophthalmol.* (2017) 101:1334–9. 10.1136/bjophthalmol-2016-309749 28232381

[28] Bell KJ Hayen A Glasziou P Mitchell AS Farris M Wright J Early CRT monitoring using time-domain optical coherence tomography does not add to visual acuity for predicting visual loss in patients with central retinal vein occlusion treated with intravitreal ranibizumab: a secondary analysis of trial data. *Retina.* (2017) 37:509–14. 10.1097/iae.0000000000001207 27548351

[29] Januschowski K Feltgen N Pielen A Spitzer B Rehak M Spital G Predictive factors for functional improvement following intravitreal bevacizumab injections after central retinal vein occlusion. *Graefes Arch Clin Exp Ophthalmol.* (2017) 255:457–62. 10.1007/s00417-016-3471-2 27632214

[30] Brogan K Precup M Rodger A Young D Gilmour DF. Pre-treatment clinical features in central retinal vein occlusion that predict visual outcome following intravitreal ranibizumab. *BMC Ophthalmol.* (2018) 18:37. 10.1186/s12886-018-0701-x 29426292 PMC5807839

[31] Scott IU VanVeldhuisen PC Ip MS Blodi BA Oden NL King J Baseline factors associated with 6-month visual acuity and retinal thickness outcomes in patients with macular edema secondary to central retinal vein occlusion or hemiretinal vein occlusion: SCORE2 study report 4. *JAMA Ophthalmol.* (2017) 135:639–49. 10.1001/jamaophthalmol.2017.1141 28492860 PMC5710260

[32] Maggio E Mete M Maraone G Attanasio M Guerriero M Pertile G. Intravitreal injections for macular edema secondary to retinal vein occlusion: long-term functional and anatomic outcomes. *J Ophthalmol.* (2020) 2020:7817542. 10.1155/2020/7817542 32104597 PMC7040414

[33] Braimah IZ Agyabeng K Amoaku WM. Efficacy of intravitreal ziv-aflibercept in patients with macular edema following retinal vein occlusion in Korle-Bu Teaching Hospital, Ghana: a retrospective case series. *Int Ophthalmol.* (2021) 41:2445–53. 10.1007/s10792-021-01799-w 33782846 PMC8238774

[34] Rayess N Rahimy E Ying GS Pefkianaki M Franklin J Regillo CD Baseline choroidal thickness as a predictor for treatment outcomes in central retinal vein occlusion. *Am J Ophthalmol.* (2016) 171:47–52. 10.1016/j.ajo.2016.08.026 27567889

[35] Suzuki M Nagai N Minami S Kurihara T Kamoshita M Sonobe H Predicting recurrences of macular edema due to branch retinal vein occlusion during anti-vascular endothelial growth factor therapy. *Graefes Arch Clin Exp Ophthalmol.* (2020) 258:49–56. 10.1007/s00417-019-04495-9 31732812

[36] Kornhauser T Schwartz R Goldstein M Neudorfer M Loewenstein A Barak A. Bevacizumab treatment of macular edema in CRVO and BRVO: long-term follow-up. (BERVOLT study: bevacizumab for RVO long-term follow-up). *Graefes Arch Clin Exp Ophthalmol.* (2016) 254:835–44. 10.1007/s00417-015-3130-z 26269374

[37] Rayess N Rahimy E Ying GS Pefkianaki M Franklin J Regillo CD Baseline choroidal thickness as a short-term predictor of visual acuity improvement following antivascular endothelial growth factor therapy in branch retinal vein occlusion. *Br J Ophthalmol.* (2019) 103:55–9. 10.1136/bjophthalmol-2018-311898 29567791

[38] Liu W Li Y Cao R Bai Z Liu W. A systematic review and meta-analysis to compare the efficacy of conbercept with ranibizumab in patients with macular edema secondary to retinal vein occlusion. *Medicine (Baltimore).* (2020) 99:e20222. 10.1097/MD.0000000000020222 32481293 PMC7249991

[39] Li F Sun M Guo J Ma A Zhao B. Comparison of conbercept with ranibizumab for the treatment of macular edema secondary to branch retinal vein occlusion. *Curr Eye Res.* (2017) 42:1174–8. 10.1080/02713683.2017.1285943 28441077

